# Assessing the efficacy of zinc oxide nanoparticles against *Penicillium expansum* by automated turbidimetric analysis

**DOI:** 10.1080/21501203.2017.1369187

**Published:** 2017-09-06

**Authors:** Davide Sardella, Ruben Gatt, Vasilis P. Valdramidis

**Affiliations:** a Department of Food Studies and Environmental Health, Faculty of Health Science, University of Malta, Msida, Malta; b Centre for Molecular Medicine and Biobanking, University of Malta, Msida, Malta; c Metamaterials Unit, Faculty of Science, University of Malta, Msida, Malta

**Keywords:** Turbidimetry, nanoparticles, *Penicillium*, postharvest, MIC, NIC

## Abstract

Public concerns about food safety have triggered a worldwide implementation of new legislations aimed at banning many of the most popular food conventional antifungal treatments. There is therefore an urgent need to identify novel and safer solutions to prevent fungal contamination of food. The antifungal effect of zinc oxide nanoparticles (ZnO NPs) against the postharvest pathogenic fungus *Penicillium expansum* has been investigated in this study. An automated turbidimetric assay, with a standard 96-well microplate, has been developed and optimised regarding the selection of the inoculum size in order to collect sequential optical density measurements. Data were processed by the updated version of the Lambert Pearson model to estimate the minimum inhibitory concentration and the non-inhibitory concentration values which were found to be 9.8 and 1.8 mM (i.e. 798 and 147 ppm), respectively. The current results show that turbidimetry is a reliable technique for assessing the antifungal activity of metal nanoparticles and that zinc oxide (ZnO) is an effective fungicide which can be potentially used to control food safety.

## 1. Introduction

*Penicillium expansum* is one of the oldest described *Penicillium* species and it has been established as the main cause of spoilage of pome fruits (Pitt and Hocking 2009). At least 17 *Penicillium* spp. have been isolated from naturally infected pome fruits with blue mould but *P. expansum* is by far the most common species (Sutton et al. 2014). Nevertheless, *P. expansum* has been isolated from a wide range of other fruits including tomatoes, strawberries, avocados, mangoes and grapes (Snowdon 1990; Pitt and Hocking 1997) indicating that it is a broad spectrum pathogen on fruits. Several chemical agents, used in storage facilities, have been developed to control this disease, such as deoxyglucose, chlorine dioxide and calcium chloride (Pitt and Hocking 2009). Compounds like benzimidazole, which effectively controlled blue mould for more than 20 years, are no longer effective due to the development of resistant populations of *P. expansum* (Sutton et al. 2014), while applications of fludioxonil, pyrimethanil and pyraclostrobin-boscalidcan still provide good control of this disease (Sutton et al. 2014). However, it has been shown that pyraclostrabin and fludioxonil are highly to very highly toxic to invertebrates, vertebrates and algae in freshwater and marine systems (Elskus 2012) while pyrimethanil is classified by the US Environmental Protection Agency as a possible human carcinogen since it produces thyroid tumours in rats (Hurley et al. 1998; Elskus 2012). Postharvest disease management is thus currently suffering on one hand, as many control strategies are not anymore effective and, on the other hand, as most of the current effective compounds have shown to be toxic and harmful to humans or to aquatic communities. Furthermore, cold storage atmosphere by itself does not prevent fruit rot since minimum temperatures for growth of *P. expansum* have been reported to be as low as −6°C, −3°C and −2°C (Brooks & Hansford 1923; Panasenko 1967; Pitt and Hocking 2009). In order to overcome these issues, it is important to explore novel antifungal agents which may replace current control strategies (He et al. 2011). Recently, metal nanoparticles have received increasing attention due to strong antimicrobial properties and low toxicity towards mammalian cells and have been already applied in food preservation, burn dressings, safe cosmetics, medical devices, water treatment and other range of products (Moritz and Geszke-Moritz 2013). Additional benefits coming from the use of inorganic nanoparticles are their improved stability in comparison with the traditional antimicrobial agents (Dutta et al. 2012) and some of them even contain mineral elements essential to human (Roselli et al. 2003; Espitia et al. 2012). The main metal and metal oxide nanoparticles are based on silver, copper, copper oxide and zinc oxide (Moritz and Geszke-Moritz 2013). Silver has been in use since time immemorial in the form of metallic silver, silver nitrate, silver sulfadiazine for the treatment of burns, wounds and several bacterial infections (Rai et al. 2009). However, silver nanoparticles have some risks as the exposure to silver can cause agyrosis (Rai et al. 2009) and it is toxic to mammalian cells (Gong et al. 2007) unless if used in minute concentrations. Copper and its compounds have also been used for centuries as effective antibacterial, antifungal and antiviral agents (Ingle et al. 2014). Copper compounds may be toxic to fish and other organisms and they may also cause environmental hazards. Therefore, direct use of copper and copper compounds in high doses should be avoided (Ingle et al. 2014).

Zinc oxide (ZnO) is an inorganic compound widely used in everyday applications. Amongst the wide range of the currently available metal nanoparticles, zinc oxide nanoparticles (ZnO NPs) possess superior durability, greater selectivity and heat resistance than others (He et al. 2011). ZnO in nanoscale found potential applications in food preservation since ZnO NPs have been incorporated in polymeric matrices in order to provide antimicrobial activity to the packaging material (Espitia et al. 2012). Additionally, ZnO NPs have also been tested as a potential coating agent on strawberries to control microbial contamination (Aponiene et al. 2015). Few studies have focused on the toxicological impact of ZnO NPs as well as studies about biotransformation and elimination routes (Espitia et al. 2012). However, ZnO is currently one of the five zinc compounds that are listed as a generally recognised as safe substance by the US Food and Drug Administration (FDA 2016).

The objective of this study was to develop an innovative quantitative rapid responses technique for assessing the antifungal activity of ZnO NPs against *P. expansum* and identifying its minimum inhibitory concentrations.

## 2. Materials and methods

### 2.1 Preparation of inocula

*Penicillium expansum*, used for this study and previously isolated from “Golden Delicious” apples, was kindly provided by the fungal collection of the Postharvest Pathology group of IRTA (Spain). Fungal spores were harvested from a 5-day old malt extract agar (Biolife, Milano, Italy) Petri dish culture by adding 10 mL of a 0.05% Tween-80 solution and by scraping off the plate’s surface with a sterile bent rod. The resulting suspension was aseptically filtered through a 4-layer sterile gauze to remove any mycelial contamination. The turbidity of the resulting suspension was adjusted to an optical density (OD) value of 0.25 at 550 nm with a colorimeter (Jensen, Northern Carolina, USA). Spores’ concentration was then determined with a haemocytometer and was found to be 2 × 10^6^ spores/mL. Two 10-fold sequential dilutions were then prepared from the neat suspension.

The choice of the proper inoculum size, in terms of best reproducibility and lowest variation during the antifungal screening upon repeated experiments, has been investigated in the beginning of this study for the nanoparticles’ concentrations from C_1_ to C_5_ (listed in Table 1) as well as for each control.

**Table 1.** List of all the tested concentrations of ZnO NPs.

	mM	ppm
C_0_	0	0
C_1_	0.5	41
C_2_	1	81
C_3_	2	163
C_4_	2.5	204
C_5_	3	244
C_6_	4	326
C_7_	5	407
C_8_	6	488
C_9_	7	570
C_10_	8	651
C_11_	9	733
C_12_	15	1221

Range starts from a concentration of 0.5 mM corresponding to 41 ppm (C_1_) up to 15 mM corresponding to 1221 ppm (C_12_).

### 2.2 Preparation of the medium

This study was carried out on semi-solid potato dextrose agar (PDA) prepared from raw potatoes in order to get a more satisfactory potato infusion than commercially dehydrated forms (Pitt and Hocking 2009). Agar content was 0.125% w/v since it has been shown to give reproducible and consistent results upon repeated experiments (Medina et al. 2012). The particular semi-solid (0.125%) agar composition has been shown to result in the development of a homogeneous hyphal network thus reducing light crossing the well in a way that is proportional to hyphal growth and, at the same time, preventing pellet sedimentation onto the bottom of the wells (Medina et al. 2012). To prepare the potato infusion, 20 g of sliced, peeled potatoes (Category: Agata, Origin: Italy) were boiled in 100 mL of distilled water for 30 min. After boiling, the potato infusion was cooled down to room temperature and potato slices were filtered through a cheesecloth decanting the effluent into the rest of the infusion. The refraction index, a measure of the total soluble solids, of the potato infusion was measured with a refractometer (ATAGO, Japan) and was found to be *n*_D_ = 1.3340 ± 0.0001 at 25°C, indicating low variation between different batches of the raw ingredients. Finally, 2 g of dextrose were added to the infusion, mixed thoroughly with agar and final volume was brought back to 100 mL with distilled water. Medium was sterilised by autoclaving at 121°C for 15 min.

### 2.3 Antifungal screening

A turbidimetric assay was performed to investigate the antifungal activity of ZnO NPs. ZnO nanopowder (<50 nm particles size, Sigma Aldrich, St. Louis, USA) was suspended into 100 mL of semi-solid PDA to reach the final concentrations illustrated in Table 1. Media with nanoparticles were then placed into an ultrasonic bath at 37 kHz sonicating frequency (Elmasonic S60, Elma, Singen, Germany) for 30 min in order to break nanoparticles’ aggregations. An additional medium without ZnO was used as a control. Hereafter, 150 μL of the medium and 20 µL of the inoculum suspension were decanted and mixed into a standard 96-well flat-bottom plate for microtitration (Thermo Scientific, Denmark). Non-inoculated media with and without nanoparticles were also prepared and used as negative controls. The plate was incubated at 25°C into a Tecan Sunrise™ microtitre reader (Tecan, Salzburg, Austria). OD was read at 600 nm automatically every 20 min without shaking. It is important to indicate that a reading at 600 nm is far away from the absorbance peak of ZnO (370 nm), so that light scattering due to Tyndall effect is negligible. Sequential OD measurements were performed to generate OD curves for each sample at least in triplicate. Each time the corresponding positive controls (C_0_) were also collected.

## 3. Calculation (quantitative assessment of the inhibitory concentrations)

For this study, the area under the *OD* versus *time* was calculated by the trapezoidal method in Microsoft Excel®. The ratios of the areas, or fractional areas (*fa*), with and without ZnO NPs, referred to as *A*_c_ and *A*_c0_, respectively, were then calculated as follows (Guiller et al. 2007):

(1) $$fa\left(c\right)=\;A_{c}/A_{c0}$$

where *c* is the concentration of ZnO. The updated version (Lambert and Lambert 2003) of the Lambert–Pearson model (LPM) based on a Gompertz function (Lambert and Pearson 2000) was then used to model the observed fractional areas:

(2) $$fa\left(c\right)=\exp\left[-{\left(\frac{c}{p_{1}}\right)}^{p_{2}}\right]$$

where, *p*_1_ is the concentration at maximum slope and *p*_2_ is a slope parameter. The regression analysis was performed by using the Generalised Reduced Gradient algorithm (Excel solver) and the least squares optimisation where the objective function is expressed as a sum of squares. The minimum inhibitory concentration (MIC), defined as the concentration above which no growth is observed relative to the control and the non-inhibitory concentration (NIC) defined as the concentration below which normal visible growth is observed (Chorianopoulos et al. 2006), were estimated from the LPM as follows (Chorianopoulos et al. 2006; Guiller et al. 2007):

(3) $$MIC=p_{1}\exp\left(\frac{1}{p_{2}}\right)$$

(4) $$NIC=\;p_{1}\exp\left(\frac{1-e}{p_{2}}\right)$$

## 4. Results and discussion

### 4.1 Choice of the inoculum size

When the three different inoculum sizes were assessed (Table 2) they were all showing consistent values, in terms of low standard deviation between replicates but a clear trend regarding the choice of the inoculum size was not evident. However, inoculum size of 10^5^ spores/mL showed the lowest standard deviation values in most of the experiments performed; therefore, it was chosen for carrying out the whole study. All the results shown in the next sections are obtained from experiments performed with this inoculum size.

**Table 2.** Values of the fractional areas for the first five different concentrations of ZnO tested with their respective controls and standard deviations (stdev) from eight replicates.

	*fa*(*c*)		*fa*(*c*)		*fa*(*c*)	
Concentration	10^4^ spores/mL	stdev	10^5^ spores/mL	stdev	10^6^ spores/mL	stdev
Ctrl (i)	3403	85	4073	**37**	4475	266
C1	3592	**12**	3961	44	4351	125
Ctrl (ii)	3403	85	4042	**13**	4475	266
C2	3400	**112**	3832	116	4364	54
Ctrl (iii)	3177	**39**	3866	142	4574	198
C3	2392	490	3293	**38**	4140	52
Ctrl (iv)	3220	26	3621	**12**	4165	194
C4	3567	28	2940	77	2608	**16**
Ctrl (v)	3182	**110**	3767	232	4070	160
C5	2229	35	2697	**34**	3104	188

In bold the lowest stdev for each inoculum size is indicated.

### 4.2 Antifungal effect of ZnO NPs

**Figure 1.**
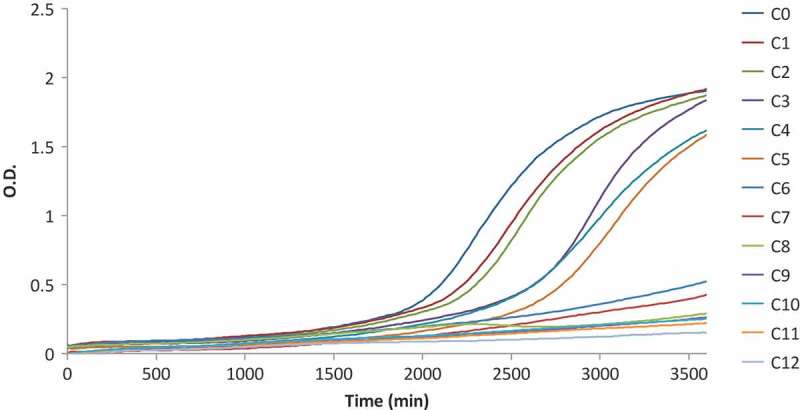
Growth curves for *P. expansum* inoculated without ZnO (C_0_) and with concentrations ranging from 0.5 up to 15 mM (C_1_–C_12_).

Following the choice of the inoculum size of *P. expansum*, the turbidimetric assay was applied to investigate the antifungal activity of ZnO NPs. Figure 1 shows the growth curves for a representative replicated experiment. The inhibitory effect of ZnO NPs (C_1_ to C_12_) against the control (C_0_) is already evident from the lowest concentration of ZnO (C_1_). Samples inoculated with C_12_ were completely inhibited and resulted in an almost flat curve. Two significant shifts in the growth trends are present between C_2_–C_3_ and C_5_–C_6_.

**Figure 2.**
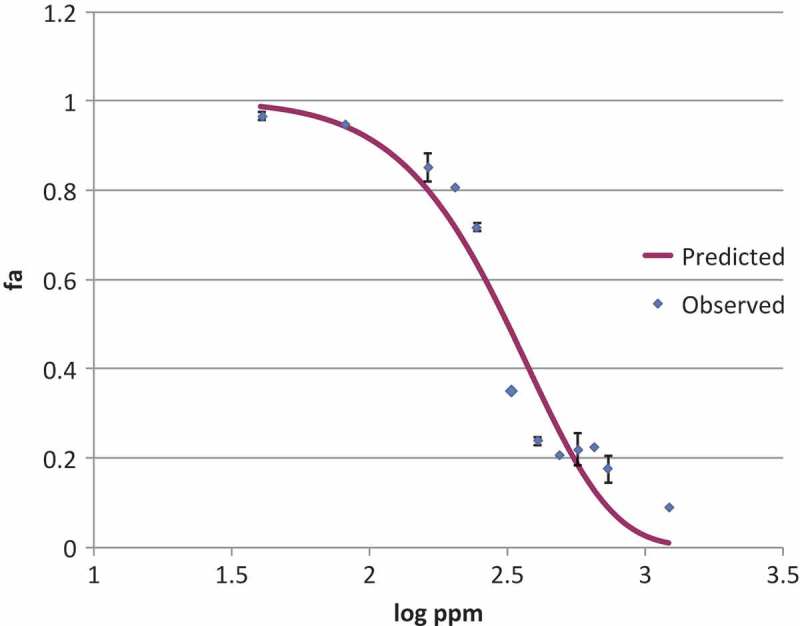
Inhibition profile of ZnO nanoparticles against *P. expansum*. Scattered points represent the averages of the observed values of estimated fractional area, continuous line represents the predicted values obtained from the LPM. The mean squared error (MSE) was 0.08.

The dependent (*fa*) and independent variables (logarithm of the ZnO concentration expressed in log ppm) of Equation 2 were plotted in a graph (Figure 2). A non-linear regression analysis of Equation 2 was performed to estimate *p*_1_ and *p*_2_ and MIC and NIC were then calculated by Equations 3 and 4. MIC and NIC were found to be 9.8 and 1.8 mM (7988 and 147 ppm), respectively.

Current results indicate that ZnO NPs with sizes <50 nm possess a significant antifungal activity against *Penicillium expansum*, the causative agent of blue mould, known as the most common postharvest decay of apples and pears (Sutton et al. 2014; Sardella et al. 2016). ZnO has already been shown to be effective against filamentous fungi in several studies (Sawai 2003; He et al. 2011; Savi et al. 2013) using conductimetry and growth diameter measurements. He et al. investigated the antifungal effect of ZnO NPs with sizes of 70 ± 15 nm in concentrations ranging from 0 up to 12 mM against *P. expansum* (He et al. 2011). Their results indicated that concentrations of ZnO NPs higher than 3 mM could significantly inhibit the radial growth of *P. expansum* onto Petri dishes, but no lower concentrations in the range between 0 and 3 mM were investigated. The current results show that concentrations even lower than 3 mM may also exhibit a clear antifungal effect for nanoparticles with sizes <50 nm. Furthermore, the MIC and NIC were estimated by performing non-linear regression analyses on the obtained data which allows for a quantitative and accurate antifungal activity assessment. Other researchers (Sawai and Yoshikawa 2004) evaluated the antifungal activity of ZnO bulk powder against *Saccaromyces cerevisiae*, *Candida albicans*, *Aspergillus* niger and *Rhizopus stolonifer* using an indirect conductimetric assay. This technique is based on the changes in electrical conductivity of an alkaline solution produced by CO_2_ absorption from fungal metabolism. Conductimetric data were processed by the authors using the growth inhibition kinetic model proposed by Takahashi (1990) for the estimation of the MIC which was reported to be ≈1.2 M, thus suggesting a lower antifungal activity of bulk ZnO formulations compared to nanoparticles presented in the current study. When Savi et al. (2013) compared the effect of different Zn-compound treatments against *Fusarium graminearum*, *Penicillium citrinum* and *Aspergillus flavus* concluded that ZnSO_4_ and Zn(ClO_4_) showed better antifungal activity than nano and bulk ZnO (Savi et al. 2013). It is therefore evident that appropriate selection of the form of ZnO or Zn salts should be made depending on the specific applications.

Nutrients’ availability plays also a fundamental role for the capacity of the fungi to recover from the antagonistic effect of the nanoparticles. The medium used in the current study, prepared from fresh potatoes, contains a richer source of nutrients than dehydrated media, as also stated by Pitt and Hocking (2009). Commercially available powders should therefore be carefully used while assessing antifungal compounds for fresh-food pathogens as they may lead to an overestimation of antifungal activity and provide non-realistic results. Future studies should be performed, in which the effect of the media on the antifungal properties of specific nanoparticles is assessed.

## 5. Conclusions

The development of a quantitative, rapid and reliable antifungal assay is nowadays crucial in food mycology as the necessity of testing novel antifungal compounds is recently arising. As a consequence, a method that can allow accurate results for filamentous fungi by automated monitoring represents an attracting option (Medina et al. 2012). In this study, a turbidimetric assay for the screening of ZnO NPs against a filamentous fungus was developed and optimised in order to obtain reliable and reproducible results. The investigation of the effect of different inoculum sizes did not show any significant difference for the use of spore concentrations between 10^4^ and 10^6^ spores/mL. Nevertheless, the 10^5^ spores/mL concentration showed the lowest variation in most of the cases. The MIC and the NIC of ZnO against *P. expansum* were determined as they are basic requirements for studying the antimicrobial activity of preservative agents and biocides (Lambert and Pearson 2000). It is demonstrated that the updated version of the LPM (Lambert and Lambert 2003) can be used to process the data obtained from the turbidimetric assay for successfully estimating the MIC and the NIC. In comparison with other methods for antifungal activity testing, the turbidimetric assay has the advantage of being monitored automatically allowing rapid multiple data collection and the opportunity of being implemented by mathematical models.
